# Enrichment of Targetable Mutations in the Relapsed Neuroblastoma Genome

**DOI:** 10.1371/journal.pgen.1006501

**Published:** 2016-12-20

**Authors:** Olivia M. Padovan-Merhar, Pichai Raman, Irina Ostrovnaya, Karthik Kalletla, Kaitlyn R. Rubnitz, Eric M. Sanford, Siraj M. Ali, Vincent A. Miller, Yael P. Mossé, Meaghan P. Granger, Brian Weiss, John M. Maris, Shakeel Modak

**Affiliations:** 1 Children’s Hospital of Philadelphia and the University of Pennsylvania, Philadelphia, PA, United States of America; 2 Memorial Sloan Kettering Cancer Center, New York, NY, United States of America; 3 Foundation Medicine, Inc., Cambridge, MA, United States of America; 4 Cook Children’s Health Care System, Fort Worth, TX, United States of America; 5 Cincinnati Children’s Hospital Medical Center, Cincinnati, OH, United States of America; Dana Farber Cancer Institute, UNITED STATES

## Abstract

Neuroblastoma is characterized by a relative paucity of recurrent somatic mutations at diagnosis. However, recent studies have shown that the mutational burden increases at relapse, likely as a result of clonal evolution of mutation-carrying cells during primary treatment. To inform the development of personalized therapies, we sought to further define the frequency of potentially actionable mutations in neuroblastoma, both at diagnosis and after chemotherapy. We performed a retrospective study to determine mutation frequency, the only inclusion criterion being availability of cancer gene panel sequencing data from Foundation Medicine. We analyzed 151 neuroblastoma tumor samples: 44 obtained at diagnosis, 42 at second look surgery or biopsy for stable disease after chemotherapy, and 59 at relapse (6 were obtained at unknown time points). Nine patients had multiple tumor biopsies. *ALK* was the most commonly mutated gene in this cohort, and we observed a higher frequency of suspected oncogenic *ALK* mutations in relapsed disease than at diagnosis. Patients with relapsed disease had, on average, a greater number of mutations reported to be recurrent in cancer, and a greater number of mutations in genes that are potentially targetable with available therapeutics. We also observed an enrichment of reported recurrent RAS/MAPK pathway mutations in tumors obtained after chemotherapy. Our data support recent evidence suggesting that neuroblastomas undergo substantial mutational evolution during therapy, and that relapsed disease is more likely to be driven by a targetable oncogenic pathway, highlighting that it is critical to base treatment decisions on the molecular profile of the tumor at the time of treatment. However, it will be necessary to conduct prospective clinical trials that match sequencing results to targeted therapeutic intervention to determine if cancer genomic profiling improves patient outcomes.

## Author Summary

Neuroblastoma is a pediatric cancer that usually affects children within the first five years of life. The survival rate for the high-risk form of the disease is 40–50%, and patients suffering metastatic recurrences have no known curative therapeutic options. Drugs targeted to specific genetic alterations in neuroblastoma may be more effective. Although neuroblastomas generally have few actionable genetic alterations at diagnosis, targetable mutations that confer therapy resistance may be selected for over time. Here, we analyzed cancer gene panel sequencing data from 151 neuroblastomas acquired at various time points during therapy to further define how the genomic landscape of neuroblastoma evolves. We found that relapsed tumors tended to have a higher frequency of mutations potentially targetable with currently available therapies, particularly in the RAS/MAPK pathway. Our data support the concept that therapeutic decisions targeting specific oncogenic mutations should be based on sequencing data obtained as close to the intervention as possible, and not be reliant on archived diagnostic material. Prospective clinical trials will be required to determine if sequencing data obtained at the time of tumor progression can lend to improved neuroblastoma patient outcomes.

## Introduction

Neuroblastoma is a cancer typically affecting young children arising from the developing sympathetic nervous system, but can occasionally occur in adolescents and adults [1]. Over half of patients have widely metastatic disease at diagnosis where survival rates are less than 50% despite intensive therapeutic regimens including chemotherapy, radiation therapy and immunotherapy [2]. There is no standard therapeutic approach for relapsed disease [3]. Recent next generation sequencing (NGS) efforts of matched neuroblastoma samples collected at diagnosis and constitutional DNAs in 373 unique subjects across four studies has clearly documented a relatively low somatic mutation rate in the protein coding portion of the genome [4–7], challenging the concept of targeting oncogenic drivers with newly developed therapeutics. The data in neuroblastoma appears to be reflective of pediatric cancers in general [8]. However, recent studies of diagnostic-relapse-normal DNA “trios” from a limited number of neuroblastoma cases has shown that the mutation rate is much higher after exposure to genotoxic chemoradiotherapy, and that there may be an enrichment of previously subclonal mutations in pathways known to be therapeutically targetable in other diseases [9–11].

To better understand and characterize the landscape of potentially actionable mutations at both diagnosis and relapse, we analyzed targeted next-generation sequencing data for 151 neuroblastomas from 138 patients that were profiled either at diagnosis, in the midst of therapy, and/or at disease relapse. Our primary aim was to retrospectively determine the frequency by which a therapeutically relevant lesion was discovered at these time points and to infer if the biopsy procedure followed by DNA sequencing could provide the potential for patient benefit.

## Results

**Fig 1:**
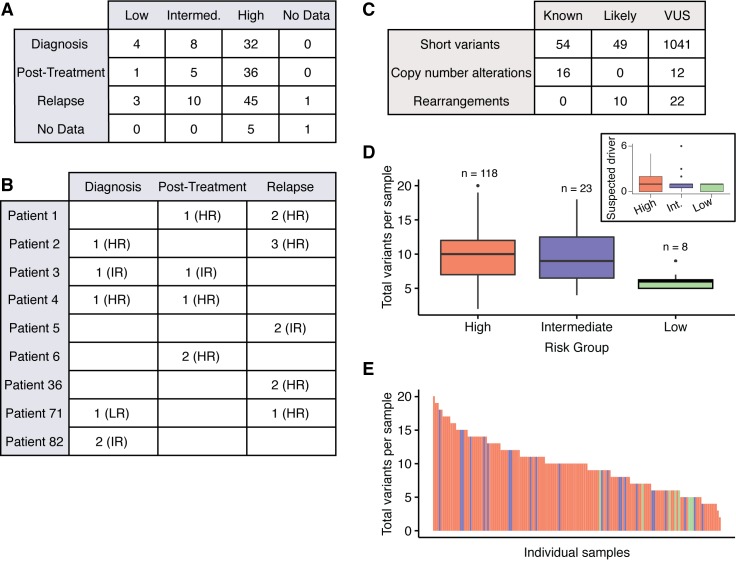
**Study cohort overview** A) Tabulation of Children’s Oncology Group (COG) risk classification and treatment time points of biopsy for 151 samples. (Intermed. = intermediate risk group) B) Number of samples taken at each treatment time point for nine patients with serial biopsies. (HR = high risk, IR = intermediate risk, LR = low risk at time of biopsy; further information in S2 Table) C) Tabulation of all variants identified (VUS: variants of unknown significance) D) Total number of variants identified per sample, stratified by COG risk group. Inset shows a similar calculation for suspected driver variants only. Heavy line represents the median of the data. “n” indicates the number of patients in each risk group. E) Total number of variants in each sample. Each bar represents an individual sample; color corresponds to risk group (red = high, blue = intermediate, green = low).

We collected sequencing data and clinical information of neuroblastoma patients whose tumor biopsies had been sent to Foundation Medicine for molecular profiling (see Methods for sample processing details). The only inclusion criterion was the availability of high-quality sequencing data, and we did not filter the cohort further based on disease stage, risk group, age, or presence of molecular lesions. We analyzed data from 151 samples from 138 individuals at various time points during treatment (44 at diagnosis, 42 after the start of treatment, 59 at relapse, and 6 at unknown time points) and with varying risk status (Fig 1A, S1 Table). Samples labeled as “diagnosis” were biopsied before the start of treatment, “relapse” samples were taken at the time of disease relapse, and “post-treatment” samples comprised refractory disease, samples collected at definitive surgery, and any other non-relapsed tumors that had been exposed to treatment (generally 3–4 rounds of chemotherapy). Nine patients had serial biopsies sent for profiling (Fig 1B, S2 Table).

Across the cohort, we identified 1204 unique variants involving 352 unique genes (Fig 1C). We define “suspected driver” variants as any short variants (single amino acid substitution or short insertion/deletion) that appear in the Catalog of Somatic Mutations in Cancer (COSMIC), any copy number alterations (CNAs) that have been shown in the literature to be known oncogenic drivers, or any type of variant that disrupts a tumor suppressor gene, falls within a known hotspot, or is a fusion involving oncogenic driver kinases. Any type of variant uncharacterized in the literature is reported as a variant of unknown significance (VUS). It is important to note that every sample in this study was taken from a tumor biopsy without a paired germline sample from the same individual. Therefore, excluding the patients for whom we have data from multiple biopsies–and in whom mutations were only identified in a subset of biopsies–we cannot rule out the possibility that any reported mutation may be from the germline genome.

We detected an average of 9.8 total variants per sample, with a range of 2–20. The number of suspected driver variants was lower, with an average of 1.28 variants per patient, and a range of 0–6. High risk tumors had, on average, a higher total number of variants (10.1 per sample for high risk vs. 6.13 per sample for low risk, P = 5.49x10^-6^) and a higher number of suspected driver variants (1.33 per sample for high risk, 0.625 per sample for low risk, P = 0.0058) (Fig 1D and 1E). There was no statistically significant difference in the number of mutations between high and intermediate risk tumors.

**Fig 2:**
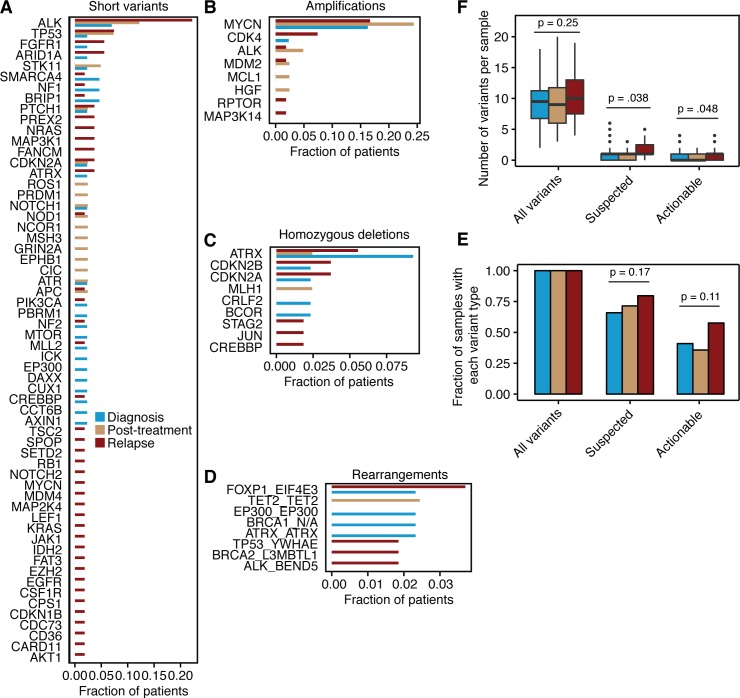
**Suspected driver variants in diagnostic, treated, and relapsed neuroblastomas** A) Frequency of all suspected driver short variants (single nucleotide variants, insertions, deletions) per gene in the entire cohort. Bars represent the number of patients with at least one lesion in a given gene, normalized to the total number of patients at each time point. Patients with multiple lesions in the same gene were only counted once per gene. B) All suspected driver amplification events with copy number >10. Bars represent the number of patients at each time point with a given gene amplification, normalized to the total number of patients at that treatment time point. C) All suspected driver homozygous deletion events. Bars represent the number of patients at each time point with a given gene loss, normalized to the total number of patients at that treatment time point. D) All suspected driver gene rearrangements and fusions. Bars represent the number of patients at each time point with a given genomic rearrangement, normalized to the total number of patients at that treatment time point. E) Number of variants in each sample, stratified by type of variant (“All variants” includes suspected driver variants as well as VUSs; “Suspected” includes suspected driver variants only; “Actionable” are suspected driver variants that have FDA approved or investigational therapy matches) and disease time point (blue, diagnosis; yellow, post-treatment; red, relapse). P values calculated using Welch’s T-test. F) Fraction of samples containing at least one variant of each type (variant types as described in (E)), stratified by disease time point. P values calculated using Fisher’s exact test.

Among the entire cohort of 151 unique neuroblastoma cases, we detected suspected driver single nucleotide variants or short insertions/deletions in 60 genes (Fig 2A); amplifications in eight genes (Fig 2B), homozygous deletions in nine genes (Fig 2C), and genomic rearrangements or fusions in eight genes (Fig 2D). *ALK* was the most commonly mutated gene, with variants occurring in 20% of patients overall (suspected driver variants in *ALK* occurred in 16% of patients). Suspected driver *ALK* variants were present in 3/43 (7.0%) of samples at diagnosis, 7/41 (17%) post-treatment samples, and in 11/54 (20%) of samples at relapse. We observed a similar trend for suspected driver short variants in other genes (41/60 genes were mutated in a higher fraction of patients after treatment than at diagnosis; P = 0.0062; Fig 2A) and for suspected driver high-level copy number amplifications (8/8 genes were amplified in a higher fraction of patients after treatment than at diagnosis; P = 0.0078; Fig 2B). However, homozygous deletions and genomic rearrangements were present at a similar frequency before and after treatment (Fig 2C and 2D).

The observation that a majority of genes were mutated or amplified in tumors that had undergone treatment led us to ask the inverse question; specifically, whether tumors that had undergone treatment were more likely to have a higher number of suspected driver variants. Although the total number of variants per sample was similar at all treatment time points (P = 0.25 between diagnosis and relapse), we found that the average number of suspected driver variants increased from diagnosis to relapse (1.1 variants per sample at diagnosis vs. 1.7 variants at relapse; P = 0.038; Fig 2E). The likelihood of a tumor to harbor at least one suspected driver variant also increased from diagnosis to relapse, although to a less significant degree (66% of tumors showed at least one variant at diagnosis, while 80% did at relapse; P = 0.17; Fig 2F).

With a relatively higher frequency of genomic alterations at relapse, it seems likely that there may be more targeted therapeutic options available for patients who have previously undergone conventional treatments. We therefore identified 40 of the 80 genes containing suspected driver variants as “potentially actionable” by annotating those that have an existing FDA approved or investigational therapy matches (see Methods and S4 Table). On average, samples taken at relapse had a higher number of potentially actionable variants (0.57 at diagnosis vs. 0.95 at relapse; P = 0.048; Fig 2E), and each relapse sample was more likely to have at least one actionable variant (41% of tumors had at least one actionable variant at diagnosis vs. 58% at relapse; P = 0.11; Fig 2F).

**Fig 3:**
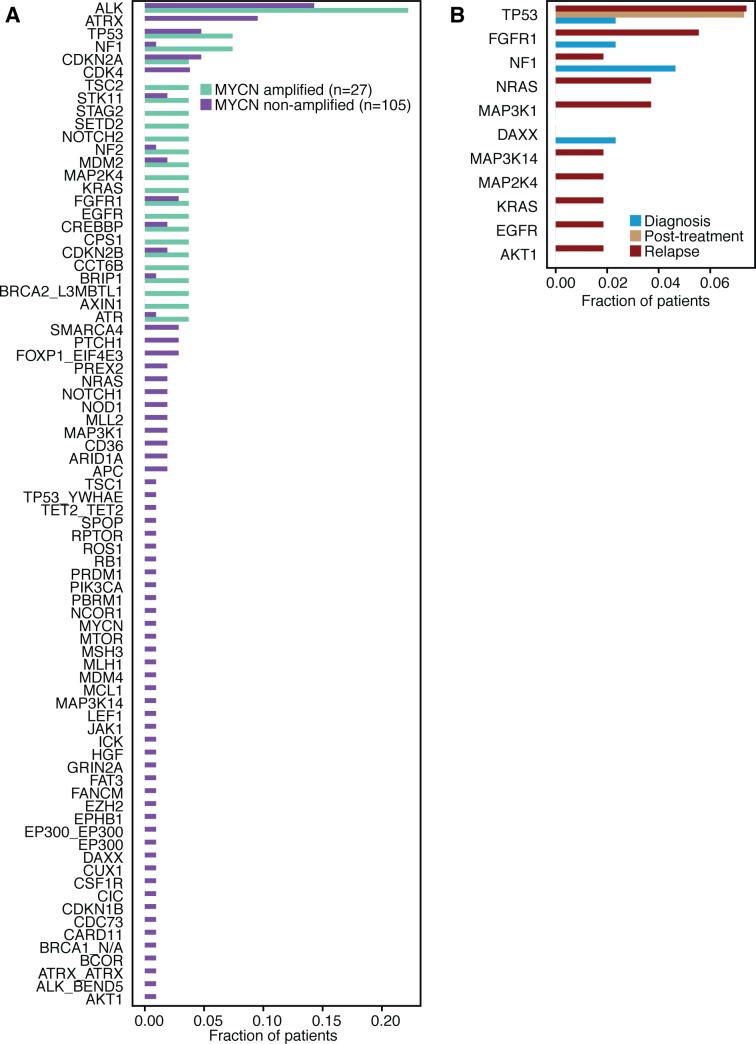
**Mutation frequencies in *MYCN* amplified and non-amplified tumors, and in the MAPK pathway** A) Frequency of suspected driver genomic alterations (short variants, copy number changes, and genomic rearrangements and fusions; *MYCN* amplification events excluded) in patients with and without *MYCN* amplification. Bars represent number of patients with at least one lesion in a given gene, normalized by the number of patients in each category. B) Percentage of patients with any suspected driver variant in the MAPK pathway at diagnosis, after treatment, and at relapse. Patients with multiple mutations in the same gene are only counted once.

We next examined trends in the data that might be of clinical relevance. First, we looked at differences in the mutational profiles between *MYCN* amplified and non-amplified tumors. *ALK* mutations were found in both *MYCN* amplified and non-amplified cases (Fig 3A), but several other variants were observed only in one context or the other (i.e. *ATRX* mutations only in *MYCN* non-amplified cases, as reported previously [4,7], and *STAG2* mutations only in *MYCN* amplified cases). The majority of these variants only appear in a single patient in the cohort, so these trends towards mutual exclusivity will need to be confirmed in large case series. We also examined the frequency of alterations in the MAPK pathway (gene set in S6 Table) at diagnosis and relapse. Fifteen unique patients harbored suspected driver alterations in the MAPK pathway at relapse, while only five tumors had a similar aberration at diagnosis (P = 0.076, Fig 3D). These data support our prior observation that mutations predicted to activate the MAPK pathway are enriched at disease relapse (Fig 3B) [9].

Nine patients had samples submitted for sequencing at various times in therapy (S2 Table). Of these, two patients did not have tumor samples with driver mutations. However, paired samples from the remaining seven patients provided insight into neuroblastoma tumor composition and evolution. Notably, we observed that definitive clonal driver mutations such as *MYCN* amplification and *ALK* mutation identified in diagnostic samples frequently persisted as drivers following exposure to chemotherapy. Interestingly, Patient 82 presented with bilateral adrenal disease, and biopsies were obtained at diagnosis from both the right and left adrenal glands. Of the 13 total variants detected in both of these samples, 10 variants were shared in common between the two tumors, suggesting that both tumors shared a common cell of origin. Patients 1, 3, 4, and 36 had multiple biopsies from the same tumor location at different time points during therapy (Patient 1: cervical lymph node metastasis at diagnosis and relapse; Patient 3, 4: adrenal gland primary tumor at diagnosis and definitive surgery; Patient 36: tibial metastasis at two relapses). Unsurprisingly, three of the four patients showed similar mutational profiles with only slight differences at each time point; however, Patient 3 had no variants in common between the two biopsies, demonstrating the potentially significant effects of spatial tumor heterogeneity and evolution. This phenomenon was further illustrated by Patient 2, who had four serial biopsies at different anatomic sites (Table 1). This patient’s diagnostic tumor contained multiple driver lesions, but some appeared clonal (heterozygous *ARID1A* mutation with allelic fraction approximating 50%), while others appeared to be present in only a subclonal fraction of cells studied (incomplete *ATRX* deletion in this male patient and inferred *FGFR1* mutation in 30% of cells). Interestingly, two unique *ALK* mutations emerged at the first and second relapse in two different metastatic locations, and in each case these were subclonal or absent at the time of final relapse following crizotinib treatment, whereas the *ATRX* and *CDK4* copy number lesions appear to have been evolutionarily selected for and were enriched in the final relapse specimen. This single case demonstrates the absolute requirement of treating physicians to understand that mutations are not binary events, and consideration of whether or not a lesion is clonal or subclonal may have major implications in the outcomes of targeted therapeutic interventions.

**Table 1:** Genetic variants from a single patient at different treatment time points. Each biopsy was at a different anatomic site. Red denotes suspected driver variants; gray denotes variants of unknown significance. Letter preceding tumor location indicates primary (P) or metastatic (M) site. Number in parentheses indicates inferred allelic fraction for mutation calls, or inferred copy number for amplification or deletion calls. See S2 Table for additional details. Note that this patient was treated with crizotinib following the 5^th^ relapse.

Patient ID 2			
Diagnosis (P, Retroperitoneum)	4^th^ Relapse (M, Neck)	5^th^ Relapse (M, Psoas)	6^th^ Relapse (M, Abdomen)
	ALK R1275Q (0.32)		
		ALK F1245V (0.30)	
ARID1A R1950Q (0.52)	ARID1A R1950Q (0.52)	ARID1A R1950Q (0.52)	ARID1A R1950Q (0.50)
ATRX loss (0.17x)		ATRX loss (0x)	ATRX loss (0x)
BCOR loss (0.35x)			
BCOR S209L (0.99)	BCOR S209L (1.0)	BCOR S209L (1.0)	BCOR S209L (0.99)
			C11orf30 A1037T (0.33)
			CD274 H233L (0.9)
CDK4 amplification (11x)	CDK4 amplification (30x)	CDK4 amplification (11x)	CDK4 amplification (82x)
CDK4 rearrangement	CDK4 rearrangement	CDK4 rearrangement	CDK4 rearrangement
EPHA5 G783A (0.69)	EPHA5 G783A (0.66)	EPHA5 G783A (0.62)	EPHA5 G783A (0.93)
		ERBB2 G549R (0.21)	
FGFR1 N546K (0.30)			FGFR1 N546K (0.25)
FGFR4 S737F (0.36)	FGFR4 S737F (0.38)	FGFR4 S737F (0.51)	FGFR4 S737F (0.32)
			IRF8 H281D (0.25)
KIT V532I (0.35)	KIT V532I (0.35)	KIT V532I (0.37)	KIT V532I (0.05)
			PARP4 rearrangement
PBRM1 Y390F (0.51)	PBRM1 Y390F (0.52)	PBRM1 Y390F (0.51)	PBRM1 Y390F (0.52)
	RICTOR K1281T (0.23)		
		SMAD2 P177R (0.25)	

Of the 138 unique patients in this cohort, 15 were reported to have received targeted therapy based on FoundationOne sequencing results and 13 had reported outcomes. Targeted treatments included ALK inhibitors, CDK4/6 inhibitors, MEK inhibitors, Raf inhibitors, mTOR inhibitors, and antibody therapies. One patient currently has an ongoing complete response, two have ongoing stable disease (>1 year and >6 months), two patients exhibited stable disease (4 months and 1 year) followed by progressive disease, and the remaining nine patients showed progressive disease despite treatment.

## Discussion

Recent papers have demonstrated that there are relatively few somatic genetic alterations in neuroblastomas at diagnosis, but also that the frequency of mutations and other genetic aberrations increases at disease relapse [9,10]. Here we performed a retrospective study of a larger cohort of 138 patients whose tumors had been biopsied and sequenced at diagnosis, second look surgery, relapse, or some combination of these, to gain a better understanding of the mutational landscape of neuroblastoma throughout treatment and disease progression. The current study provided validation of our original observation that relapsed high-risk neuroblastoma harbor an increased mutational burden [9]. Gene panel sequencing is not designed to infer mutational burden, but our data are consistent with an enrichment of mutations in known cancer driver genes at relapse, consistent with clonal evolution under the selective pressure of chemoradiotherapy. We also showed that the prevalence of “potentially actionable” mutations increased at relapse. Our data provide support for the hypothesis that the neuroblastoma genome evolves under the selective pressure of chemoradiotherapy [9,10], and It is necessary to understand the *current* genomic landscape of a tumor rather than that at diagnosis in order to make the most informed treatment decisions. Thus, the clinical practice of acquisition and analysis of relapsed tumor material, with a view to designing prospective clinical trials to determine the impact of matched target therapy on patient outcomes should be encouraged.

In parallel with several recent reports of prospective collection of tumor genomic data using gene panel [12] or whole exome [13–15] sequencing across pediatric cancer, with or without transcriptome sequencing and at diagnosis or relapse, a few general conclusions are apparent. First, while not evaluable in this retrospective study, the prospective studies noted above all clearly demonstrate that the acquisition of high dimensional genomics data from pediatric cancers is feasible, and to date has been demonstrated to be safe to incorporate into clinical practice. This is relevant for this retrospective study of neuroblastoma because the majority of relapses in this disease are in the bone or bone marrow compartment, and often require skilled interventional radiologists and pathologists to safely access, process and prepare often small samples for sequencing. Second, together the data support the conclusion that a significant proportion of patients at relapse will have a potentially actionable oncogenic driver lesion. In this study, 58% of subjects studied at relapse showed a somatic mutation in one of several genes that are major targets for drug development in other cancers, consistent with other reports [12,14,15]. Third, this study did not study the germline genome, but certainly many of the mutations identified in this study have the potential to be germline [16], and recent studies have shown that up to 10% of pediatric patients harbor likely relevant germline mutations in known predisposition genes [12–14,17]. Paired germline and somatic DNA sequencing should be considered in the genetic evaluation of pediatric cancer subjects. Finally, our and recent studies together highlight the current dismal reality that, at present, pediatric cancer sequencing efforts are rarely leading to patient benefit. The reasons for this are multifactorial and applicable to cancer in general, such as lack of adequate mutation-drug matches for many driver lesions. Further, there is a need to look beyond mutations in the coding genome, and forays into clinical epigenomic profiling and mRNA profiling may yield other targetable genomic alterations. However, there are also likely childhood cancer specific explanations as well, such as lack of pediatric dosing information and perhaps some degree of risk aversion on the part of physicians and industry partners to expand targeted therapy options in this population of children who most likely will die from their disease. Taken together, recent efforts in next generation sequencing of pediatric cancer highlight the potential clinical significance of these efforts, but more importantly the urgent need to credential precision medicine approaches to childhood cancer in well designed prospective clinical trials. Such clinical trials are currently underway, and are investigating mutations not only through next-generation sequencing of tumor samples, but also through collecting and sequencing cell free DNA and circulating tumor DNA [18,19].

## Materials and Methods

### Ethics statement

This research study was approved by the Children's Hospital of Philadelphia Institutional Review Board (IRB 14–011037). A waiver of consent/assent was granted per 45 CFR 46.116(d) [because the research involved no more than minimal risk and could not practicably be carried out without a waiver].

### Patient cohort

The inclusion criteria for this study were a diagnosis of neuroblastoma and availability of targeted sequencing data from tumor DNA through Foundation Medicine, using the FoundationOne or FoundationHeme panels (see below). We included a total of 151 samples from 138 unique individuals sampled from 0 to 67 years (all but one patient were diagnosed before age 25). The samples fell into the following categories: 44 at diagnosis, 36 at definitive surgery after initial induction chemotherapy, six from tumors refractory to induction chemotherapy or at delayed surgery, and 59 at relapse. Time points were unknown for a further six samples. For simplicity, unless otherwise specified in the manuscript, we grouped samples into the following categories: diagnosis, relapse, and post-treatment, the latter comprising of definitive surgery, refractory, and post-therapy. Nine of the 138 patients had multiple samples tested at different time points in their therapy. The group of 151 tumors included 13 such samples.

A total of 118 samples were collected from “high-risk” patients as defined by the Children’s Oncology Group, 23 from “intermediate risk”, eight from “low risk”, and two patients were not classified. At the time the samples were analyzed, 130/151 had received some sort of therapy, at least one of: cytotoxic chemotherapy, radiation therapy, MIBG treatment, anti-GD2 immunotherapy, or isotretinoin therapy. Complete information on the patient cohort can be found in S1 Table.

### DNA extraction and panel sequencing

All details of sequencing and data processing can be found in Frampton, et al [20]. Foundation Medicine determines percent tumor nuclei through histopathological review. These results can be found in S5 Table.

### FoundationOne and FoundationOne Heme panels

FoundationOne and FoundationOne Heme are pan-cancer tests. FoundationOne interrogates the entire coding sequence of 315 cancer-related genes plus select introns from 28 genes often rearranged or altered in cancer. The FoundationOne Heme DNA panel interrogates the entire coding sequence of 405 genes and selected introns of 31 genes involved in rearrangements. These genes are known to be somatically altered in human solid cancers based on recent scientific and clinical literature. Each gene in the panel is sequenced to a typical median depth of 500X. Of the 151 tumor samples included in this study, 63 were processed using the FoundationOne panel (46 using the T5a baitset, and 17 using the T7 baitset), and 88 were processed using FoundationOne Heme (24 with the T6b baitset, and 64 with the D2 baitset). All sequenced genes are listed in S3 Table. Foundation Medicine returns calls for mutations, copy number alterations, and rearrangements in spreadsheet form. We imposed a constraint on copy number changes, and only considered gains of copy number >10 in our final analysis to rule out false positive calls that may result from aneuploidy and not focal amplification events (S1 Fig).

### Classification of “potentially actionable” variants

A search on clinicaltrials.gov was performed (in August 2016) for each of the 80 genes containing suspected driver variants. A gene was considered “potentially actionable” if a mutation in the gene was an indication for assigning a patient to an arm of a clinical trial. We identified 40 such genes.

### Statistical analyses

Association between binary variables was assessed using Fisher’s exact test. Association between continuous variables was assessed using Welch’s T test. All analyses were performed in the R statistical language. All code is available at https://bitbucket.org/opadovan/nb_fm_public.

### Data access

All the data in this manuscript are supplied in S1 Table.

## Supporting Information

S1 Fig Copy number cutoff determination. (PDF)

S1 Table Complete data. Column names are as follows: Patient.ID (unique identifier for each patient), Sample.ID (unique identifier for each sample sent to Foundation Medicine), Primary_Relapse (disease state at time of sample collection), Has.Multiple.Samples (whether we collected multiple samples from the same patient), Coverage (average sequencing depth of coverage as reported by Foundation Medicine), Event.Category (type of variant; “known” and “likely” are considered “suspected drivers”, “ambiguous” and “unknown” are variants of unknown significance), Lesion (genetic variant, allele fraction, number of supporting reads), Gene (gene containing the variant), Event (type of variant), Age.at.Diagnosis (age of patient in months), Stage (neuroblastoma stage from the International Neuroblastoma Staging System), COG.Risk.Group (risk categorization from the Children’s Oncology Group), MYCN.Gene.Status (MYCN gene amplification status), DNA.Index (tumor cell ploidy), Pathology.Grade (histology classification), Therapy.Recieved (whether patient had received therapy at time of biopsy), HR.induction.chemo (high-risk induction chemotherapy), HR.consolidation.chemo (high-risk consolidation chemotherapy), Radiation.therapy (radiation therapy), MIBG.therapy (metaiodobenzylguanadine therapy), Isotretinoin.therapy (isotretinoin therapy), Anti.GD2.immunotherapy (antibody therapy targeting the GD2 protein), IR.chemo (intermediate risk chemotherapy), Targeted.Treatment.FM (whether patient received targeted treatment based on Foundation Medicine sequencing results), Response.FM.Based.Therapy (how patient responded to targeted therapy based on Foundation Medicine sequencing results: PD = progressive disease, SD = stable disease, PR = partial response, CR = complete response), Duration.Response (duration of patient’s response to therapy based on Foundation Medicine sequencing results). No data: data was not made available. N/A, NA: Not Applicable. Unknown: Physician did not have data.
(XLSX)

S2 Table Paired samples. Data from nine unique subjects with paired samples available for analysis. Suspected driver variants are highlighted in red, and variants of unknown significance are highlighted in grey. Numbers following mutation calls indicate inferred allelic fraction and read depth support for the call, respectively. Number following amplification events indicate inferred copy number and number of exons showing amplification. See text for additional details.
(XLSX)

S3 Table FoundationOne gene lists. Complete list of all sequenced genes in each FoundationOne test. Second tab indicates which test(s) were used for each patient.
(XLSX)

S4 Table Potentially actionable genes. All genes containing suspected driver variants and our call of whether the gene is potentially actionable. See Methods for more details.
(XLSX)

S5 Table Percent tumor nuclei. Percentage of tumor nuclei in each sample, as calculated by Foundation Medicine. NA, percentage unavailable; DNA, extracted DNA was sent for testing so there was no cellularity estimate.
(XLSX)

S6 Table KEGG MAPK gene set. (XLSX)
