# Supplementary material for: Enrichment of Targetable Mutations in the Relapsed Neuroblastoma Genome
Source: PLoS Genet. 2016 Dec 20;12(12):e1006501. doi: 10.1371/journal.pgen.1006501 (PMC5172533; doi:10.1371/journal.pgen.1006501)
Supplement: S1 Fig — (PDF) [file pgen.1006501.s001.pdf]

Supplementary Figure 1

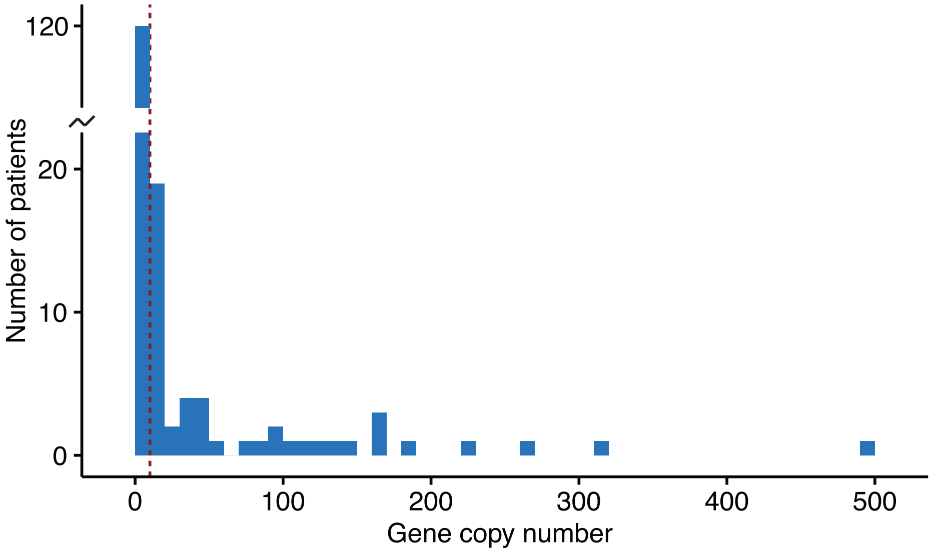

Supplementary Fig. 1. Copy number cutoff determination
Histogram of copy numbers for all copy number amplifications, of known and unknown significance. Red dashed line is at copy number 10. We disregard all amplification calls with copy number less than 10.
